# Development and optimization of human T-cell leukemia virus-specific antibody-dependent cell-mediated cytotoxicity (ADCC) assay directed to the envelope protein

**DOI:** 10.1128/jvi.02268-24

**Published:** 2025-03-28

**Authors:** Cynthia A. Pise-Masison, Mohammad Arif Rahman, Daniel C. Masison, Anna Gutowska, Ramona Moles, Massimiliano Bissa, Sarkis Sarkis, Luca Schifanella, Tongqing Zhou, Jennifer Jones, Steve Jacobson, Genoveffa Franchini

**Affiliations:** 1 Animal Models and Retroviral Vaccines Section, Basic Research Laboratory, Center for Cancer Research, National Cancer Institute, National Institutes of Health 3421 , Bethesda, Maryland, USA; 2 Laboratory of Biochemistry and Genetics, National Institute of Diabetes and Digestive and Kidney Diseases, National Institutes of Health 35043 , Bethesda, Maryland, USA; 3 Vaccine Research Center, National Institute of Allergy and Infectious Diseases, National Institutes of Health 35037 , Bethesda, Maryland, USA; 4 Translational Nanobiology Section, Center for Cancer Research, National Cancer Institute, National Institutes of Health 272101 , Bethesda, Maryland, USA; 5 Viral Immunology Section, Neuroimmunology Branch, National Institute of Neurological Disorders and Stroke, National Institutes of Health 35046 , Bethesda, Maryland, USA; St. Jude Children's Research Hospital, Memphis, Tennessee, USA

**Keywords:** HTLV-1, ADCC, CD16, adaptive immune response, natural killer cells, Fc receptors

## Abstract

An estimated 10–20 million people worldwide are infected with the deltaretrovirus human T-cell leukemia virus type 1 (HTLV-1). Although most infected individuals remain asymptomatic, some progress to develop the fatal and debilitating disease adult T-cell leukemia/lymphoma (ATLL) or HTLV-associated myelopathy/tropical spastic paraparesis (HAM/TSP) or develop a plethora of other inflammatory disorders. In addition, HTLV-1 infection is associated with immunosuppression and a shorter lifespan. Although a protective role for neutralizing antibodies has been suggested, the role of non-neutralizing antibody-dependent cell-mediated cytotoxicity (ADCC) remains unclear, largely because an assay to measure this response has not been established. Here, we developed a high-throughput flow cytometry-based assay system to measure HTLV-1 envelope-specific ADCC. We used a natural killer cell-resistant T-lymphoblastoid cell line stably expressing the green fluorescent protein GFP to construct a target cell line expressing HTLV-1 envelope protein and using monoclonal antibodies and plasma samples from HTLV-infected or uninfected individuals, validating the specificity and sensitivity of the assay. We detected high ADCC activity in samples from HTLV-1-infected humans. In the plasma of experimentally infected macaques, ADCC activity was measured and a correlation between ADCC activity and HTLV-1 envelope antibody titers was observed. Further, we observed a significant increase in ADCC titer over time; as HTLV-1 infection persists, a higher ADCC response is generated, potentially influencing disease outcome. ADCC titer in HTLV-1-infected macaques also positively correlated with FLT3LG, IL-17F, CD4^+^ T cells, and lymphocytes but negatively correlated with monocyte frequency and classical monocyte frequency. In conclusion, these findings detail the generation of a cell line that enabled development of an HTLV-specific ADCC assay, which can be employed in large clinical studies as well as research involving humans or non-human primates.

**IMPORTANCE:**

This approach measures human T-cell leukemia virus (HTLV)-specific envelope antibody-dependent cell-mediated cytotoxicity responses, provides a critical tool to investigate the role of envelope-specific binding antibodies in the immune control of HTLV infection and pathogenesis, and may help guide the development of both therapeutic and preventative vaccine approaches.

## INTRODUCTION

Although human T-cell leukemia virus type 1 (HTLV-1) elicits strong antibody and cellular immune responses, it establishes a lifelong, persistent infection. Significant progress has been made in understanding the molecular mechanisms of viral gene regulation and transmission (1, 2), but less progress has been made in understanding the host immune response to the virus and the development of effective therapeutic approaches. It is therefore necessary to focus on the immune responses against HTLV infection in order to develop both treatments and an efficacious vaccine.

Both anti-viral antibody and cell-mediated responses against the virus are detected within weeks after exposure to HTLV (3, 4). Within the first month of infection, antibodies to the viral structural Gag protein and the Envelope (Env) appear, and in half of all infected people, there is a detectable response to the viral transcriptional activator protein Tax (5). Approximately one in ten infected individuals develop antibodies to the antisense protein HBZ (6, 7), and a negligible number of individuals have detectable antibodies to Orf-I or Orf-II proteins (8, 9). Studies suggest that HTLV antibody titers to Gag and Env are significantly higher in HTLV-associated myelopathy/tropical spastic paraparesis (HAM/TSP) patients than in asymptomatic carriers, and antibody titers for Gag, Env, and Tax were higher in HAM/TSP compared to adult T-cell leukemia/lymphoma (ATLL) patients (10, 11). Furthermore, one study suggested a positive correlation between antibodies to anti-HTLV antigens and viral DNA level (plasma viral load; PVL), and a second study suggested a correlation between anti-HTLV Envelope antibodies and PVL in asymptomatic carriers but not HAM/TSP patients (12, 13). In contrast, a more recent study found no correlation between PVL and anti-HTLV-gp46 Envelope antibody levels (14). Nonetheless, the impact of anti-HTLV antibodies on disease progression remains incompletely understood.

Monoclonal antibody (mAb) binding to virus-infected cells induces death through both direct and indirect mechanisms. Antigen-specific antibodies can not only directly neutralize the virus but also trigger adaptive immune responses. Indirectly, antigen-specific antibodies can generate complement-mediated cytotoxicity, antibody-dependent cellular phagocytosis (ADCP), trogocytosis, and antibody-dependent cell-mediated cytotoxicity (ADCC) (15). ADCC is a key host defense against cell-associated viral infection (16–18). This adaptive immune response is largely mediated by natural killer (NK) cells through the CD16 (FcgRIII) receptor that binds the Fc portion of IgG antibodies bound to virus-infected cells. Receptor:antibody binding on NK cells triggers the release of contents from cytotoxic granules that invade and kill target cells by apoptosis (19). Thus, ADCC has the potential to kill HTLV-1-infected cells.

Currently, there is no reagent available that allows measurement of HTLV-1 envelope-specific antibodies mediating NK cell killing. To overcome this limitation and to measure ADCC using flow cytometry, we modified NK cell-resistant CEM cells expressing intracellular green fluorescent protein (GFP) to express the HTLV-1 envelope protein. We used this new cell line to assess the Fc-mediated functions of antibodies present in the plasma of HTLV-1-infected individuals or experimentally infected macaques. We demonstrate that antibody-specific killing can be detected using this new cell line, which stably expresses the HTLV-1 envelope protein on its surface. We further observed a positive correlation between ADCC titer and virus-specific antibody response, suggesting that a higher humoral response against the virus will generate higher ADCC responses. The ADCC response positively correlated with plasma levels of FLT3LG, IL-17F, total lymphocyte counts, and CD4^+^ T-cell counts, indicative of crosstalk among these cytokines and immune cells. Thus, this assay will allow the evaluation of both envelope antibodies and NK cell function in HTLV-1-infected individuals or experimentally infected animal models and will be instrumental for assessing the immunogenicity of preventative or therapeutic approaches against HTLV-1 infection.

## RESULTS

### Construction and characterization of the CEM-NK^R^-ENV1 cell line

**Fig 1:**
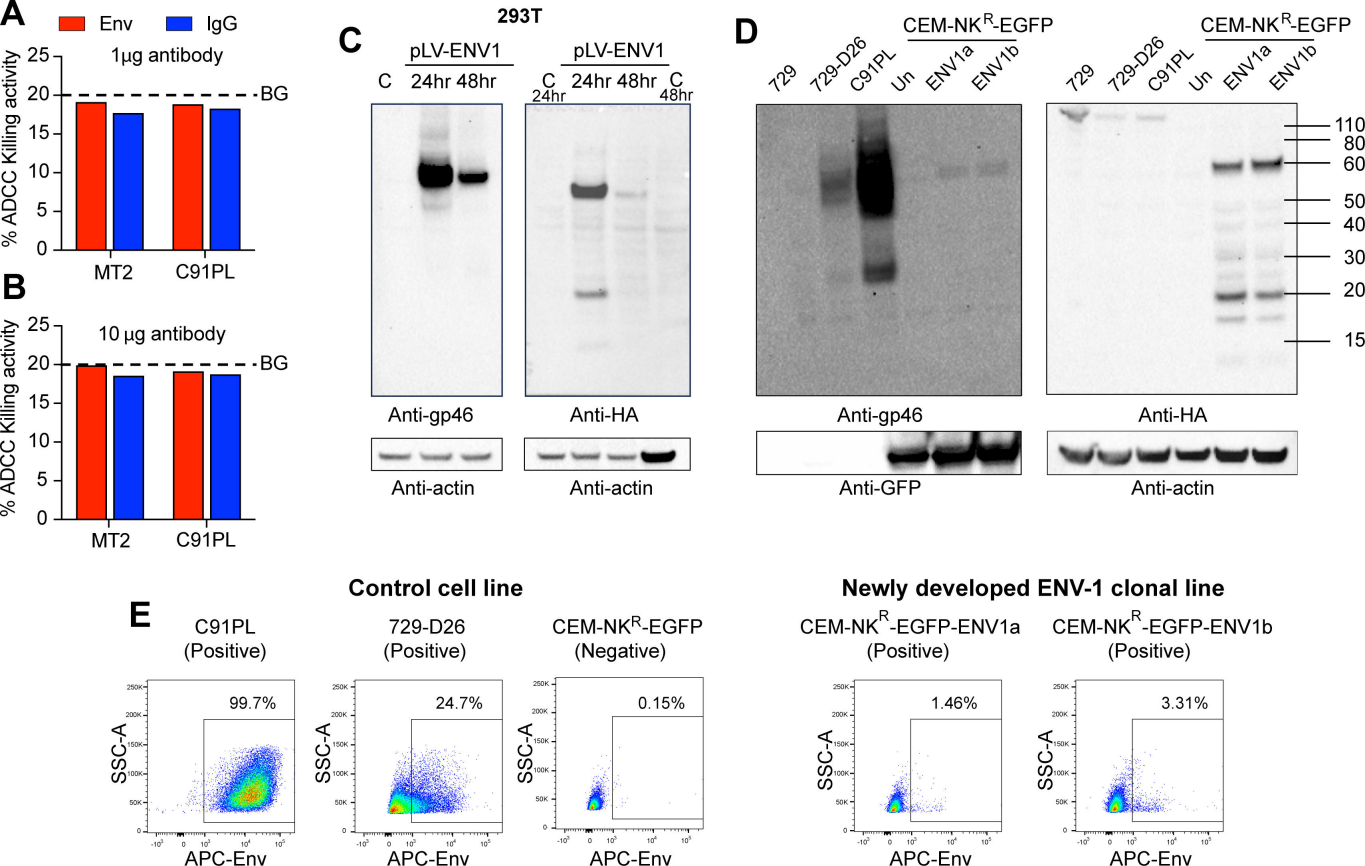
Evaluation of HTLV-1 envelope-specific ADCC killing of established cell lines and characterization of new target cell line. (**A and B**) The HTLV-1 transformed, infectious, virus-producing cell lines MT2 and C91PL were labeled with cytoplasmic dye CFSE and membrane dye PKH26. Labeled cells were used as targets in a flow cytometry-based ADCC killing assay (see Materials and Methods). Graphed is the killing activity (percent) for MT2 and C91PL at 1 µg or 10 µg of the HTLV-1 monoclonal envelope antibody PRH7a. Background levels are indicated with a dotted line. Construction of CEM-NK^R^-ENV1-EGFP cells. (**C**) Representative western blot analysis of the HTLV-1 envelope protein (anti-gp46, clone 65/6C2.2.34 Creative Biolabs, left or anti-HA, right) expression after transient transfection of the expression construct, pLv-ENV1, or control empty vector, labeled C, in 293T cells. (**D**) Stable CEM-NK^R^-ENV1-EGFP clones (clonal line 1, ENV1a and clonal line 2, ENV1b) were selected, and expression of HTLV-1 envelope protein was assessed by western blot using anti-gp46 (left, clone 65/6C2.2.34 Creative Biolabs) or anti-HA (right). Whole cell extracts for the HTLV-1 positive cell lines C91PL and 729-D26 were used as controls for HTLV-1 envelope expression, and 729 and CEM-NK^R^-EGFP (un) were used as negative controls. Anti-actin and anti-GFP antibodies were used for loading controls. (**E**) Representative flow cytometry plots for envelope expression (APC-Env) vs. cell size (SSC-A) are shown. A box around the envelope positive cells is shown on each plot.

Currently, ADCC antibody titers in plasma from HTLV-infected individuals are measured using HTLV-1 transformed cell lines, particularly MT2 cells (20–24). Because MT2 and C91PL cells are sensitive to direct NK cell killing in the absence of anti-envelope antibodies, the ADCC response measured by using these cell lines might not be antibody-specific. We tested monoclonal antibody (PRH7a)-induced ADCC killing on both HTLV-1 transformed MT2 and C91PL cell lines (Fig. 1A and B). No specific antibody-mediated killing of MT2 or C91PL cells above background levels (in the absence of PRH7a) was observed with either 1 µg or 10 µg of HTLV-1 envelope-specific monoclonal antibody. As HTLV-1 transformed MT2 and C91PL cells are sensitive to direct NK cell lysis, the lack of detectable specific ADCC activity could be due to the high background levels of cell lysis. In addition, MT2 and C91PL cells produce infectious virus that can bind to envelope antibody, which might inhibit antibody-dependent killing. Since MT2 and C91PL cells can be killed by NK cells in the absence of antibody, the need to titrate each peripheral blood mononuclear cell (PBMC) effector cell sample against the HTLV transformed target cells to establish background makes it difficult to measure ADCC using these cells. In addition, it has been shown that ADCC assays using HIV-infected primary target cells and HIV-infected CEM NK-resistant target cells show distinct ADCC responses (25). Furthermore, in a SIV macaque model, ADCC responses were detected in 28 out of 28 vaccinated macaques using coated target cells; however, virus-infected cells were only able to detect ADCC in 9–16 out of 28 macaques depending on the virus used for infecting the cells (26), suggesting that virus-infected target cells can express different surface antigens that can influence ADCC results.

We aimed to develop a reliable and rapid assay to measure gp46-mediated ADCC activity that would avoid antibody-independent NK cell killing. First, we cloned the HTLV-1A envelope cDNA with a carboxy-terminal HA-tag into a lentiviral vector with a puromycin selectable marker and detected envelope expression in whole cell lysates from transiently transfected 293T cells (Fig. 1C; Fig. S1A). This vector was used to produce lentiviral stocks for transduction of CEM-NK^R^-EGFP cells. CEM-NK^R^-EGFP cells were shown to be resistant to NK cells in the absence of antigen-specific antibody binding (27). Puromycin was used to establish stable cell lines, and envelope expression was measured using both anti-HTLV gp46 envelope and anti-HA antibodies. Both unprocessed gp68 and cleaved gp21 envelope proteins were detected by western blot using anti-HA antibody in two independent CEM-NK^R^-ENV1-EGFP cell lines but not in the CEM-NK^R^-EGFP parental cells, HTLV-1-infected C91PL T cells or 729 B cells (729-D26), or uninfected parental 729 B cells (Fig. 1D; Fig. S1B). The HTLV envelope protein (full length and processed) was detected in all HTLV-1-infected cell lines using anti-gp46 antibody but at varying levels, with C91PL cells having the highest expression and CEM-NK^R^-ENV1-EGFP cell lines having the lowest expression. HTLV-1 envelope was not detected in uninfected controls (Fig. 1D; 729, CEM-NK^R^-EGFP Un). Anti-actin and anti-GFP antibodies were used for loading controls. Next, we used flow cytometry to assess the surface expression of HTLV-1 envelope on the CEM-NK^R^-ENV1-EGFP cells compared to C91PL, 729-D26, or CEM-NK^R^-EGFP cells. As observed in the protein lysates (Fig. 1D), CEM-NK^R^-ENV1-EGFP cells had detectable but low surface expression compared to 729 wild type (WT) or C91PL (Fig. 1E). Of note, unlike what is seen in T cells from infected individuals, proviral copies of HTLV-1 in MT2 (11.7 copies/cell), C91PL (20 copies/cell), and 729-D26 (9.3 copies/cell) are greater than one copy per cell, giving rise to higher than *in vivo* levels of HTLV-1 protein expression (Table S8 and reference 28). With the aim to enrich for envelope surface expression on CEM-NK^R^-ENV1-EGFP cells, we used labeled PRH7a antibody for FACS sorting of the cells. However, after several attempts to enrich for envelope surface expression, we were unsuccessful, perhaps due to turnover of envelope protein, density of envelope expression on each cell, or epitope availability.

### High-throughput flow cytometry-based ADCC assay

**Fig 2:**
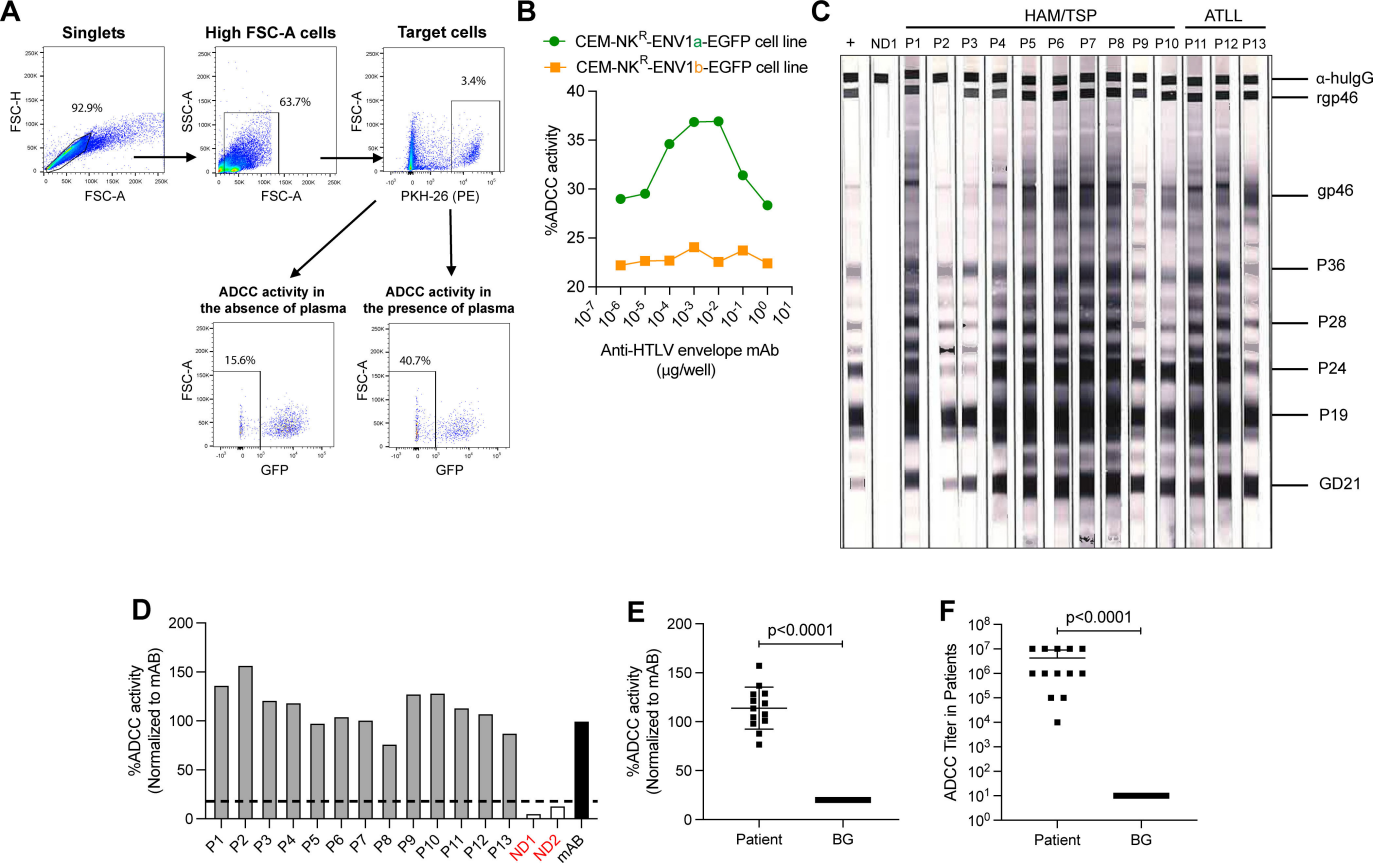
Gating strategy and evaluation of ADCC killing in patient plasma. (**A**) The flow cytometry gating strategy for determining ADCC killing activity based on loss of GFP from the PKH-26 stained target cell in the presence or absence (background activity) of plasma. (**B**) ADCC activity against HTLV-1 envelope using monoclonal antibody (PRH7a) and CEM-NK^R^-ENV1a-EGFP (green) or CEM-NK^R^-ENV1b-EGFP (orange) cell lines as target cells. (**C**) A western blot analysis to determine seroreactivity to HTLV-1 proteins for plasma samples from HAM/TSP, ATLL, and uninfected (ND1) individuals. (**D**) The percentage of ADCC killing activity in the plasma for each HTLV-1-infected or uninfected (ND1 and ND2) individual was graphed. Activity was normalized to the monoclonal envelope antibody PRH7a, set to 100%. The dotted line indicates the background level. (**E and F**) Comparison of the percent (**E**) ADCC activity or (**F**) ADCC antibody titer in the plasma of HTLV-1-positive individuals and background (BG). Data shown in (**E and F**) were analyzed with two-tailed Mann-Whitney test. Horizontal and vertical bars denote mean and SD, respectively.

Since no specific ADCC activity was detected using MT2 or C91PL (Fig. 1A and B), we tested CEM-NK^R^-ENV1-EGFP cell lines in an ADCC assay. The plasma membranes of CEM-NK^R^-ENV1-EGFP cells, which constitutively express GFP, were labeled with PKH26 and used as target cells in a flow cytometry-based ADCC assay. Plasma was incubated with the target cell for 1 hour to allow the envelope-specific antibodies to bind to the target cells. The assay was performed in a 96-well plate, with each well receiving 10,000 target cells and 500,000 PBMCs from uninfected healthy human individuals as effector cells. Effector cells were cultured with target cells at a 50:1 E/T ratio for 2 hours prior to measuring ADCC activity. Target cells were gated as PE-positive cells. PE-positive target cells that lost GFP expression were considered lysed cells. Antibody-specific killing (ADCC) was measured by subtracting killing in the presence of antibody from background (Fig. 2A). ADCC titer was measured by serial dilution of plasma in a 96-well plate; the first well received a 1:10 dilution of plasma that was then diluted 10-fold for the next seven rows. The lowest dilution of plasma at which ADCC killing can be detected is referred to as the ADCC titer. The ADCC endpoint titer is defined as the reciprocal dilution at which the percentage of ADCC killing was greater than the mean percentage of killing of the negative control wells containing medium, target, and effector cells, plus three standard deviations (29–32). CEM-NK^R^-ENV1-EGFP cell lines ENV1a and ENV1b were labeled with PKH26 and used as target cells with and without the different concentrations of envelope monoclonal antibody PRH7a. We observed that CEM-NK^R^-ENV1a-EGFP cells were more sensitive to anti-envelope-specific NK cell lysis. No such specific lysis was measured when CEM-NK^R^-ENV1b-EGFP cells were used as targets (Fig. 2B). The CEM-NK^R^-ENV1b-EGFP cells were also tested with animal plasma samples that showed specific ADCC killing with target CEM-NK^R^-ENV1a-EGFP cells and observed no killing (data not shown). Though CEM-NK^R^-ENV1b-EGFP cells express higher gp46 antigen and antibody binds to these cells, most probably the Fc portion of the bound antibody is not readily available to interact with NK cells and no killing was observed. Thus, the CEM-NK^R^-ENV1a-EGFP cell line was used in further studies.

Next, to evaluate whether this ADCC assay can specifically detect Fc function in patients living with HTLV-1, we tested the plasma from HTLV-1-infected individuals and healthy donors. Information regarding disease status, proviral load (viral copy number), and antibody titers are shown in Table 1. All plasma samples from HTLV-1-infected patients showed high seroreactivity to HTLV-1 antigens (Fig. 2C) and high envelope antibody titers as measured by enzyme-linked immunosorbent assay (ELISA) (Table 1). These plasma samples were then used to measure envelope-specific ADCC. ADCC activity was normalized to activity induced by monoclonal antibody to HTLV-1 envelope set to 100%. Plasma from HTLV-1-infected patients had >76% envelope-specific ADCC activity (Fig. 2D; Table 1). We furthermore observed significant ADCC activity and antibody titer compared to background levels (Fig. 2E and F), suggesting that this cell line was able to detect ADCC activity in the patients. Interestingly, plasma from P2 had ADCC activity and a high envelope antibody titer by ELISA but did not detect envelope by western blot (Fig. 2C), suggesting that this patient’s plasma may have ADCC directed to a conformational envelope epitope. Of note, we did not find a correlation between ADCC activity and proviral load or ADCC antibody titer and envelope antibody titer. The lack of correlation might be due to the majority of patients having a relatively high proviral load (Table 1). More studies are needed to determine what parameters influence ADCC activity and ADCC titer in patients.

**TABLE 1:** Patient information^*a*^

Sample	Disease status	PVL	ENV titer	ADCC titer	Normalized killing
P1	HAM/TSP	18.62	1 × 10^8^	1 × 10^6^	136.698984
P2	HAM/TSP	27.44	1 × 10^8^	1 × 10^6^	157.015786
P3	HAM/TSP	8.97	1 × 10^8^	1 × 10^6^	121.292365
P4	HAM/TSP	21.33	1 × 10^8^	1 × 10^6^	118.827738
P5	HAM/TSP	20.16	1 × 10^8^	1 × 10^6^	97.8790727
P6	HAM/TSP	2.27	1 × 10^8^	1 × 10^6^	104.538594
P7	HAM/TSP	27.12	1 × 10^7^	1 × 10^6^	101.049253
P8	HAM/TSP	20.44	1 × 10^9^	1 × 10^6^	76.6028281
P9	HAM/TSP	10.75	1 × 10^8^	1 × 10^6^	127.925747
P10	HAM/TSP	37.34	1 × 10^8^	1 × 10^6^	128.780917
P11	ATLL	2.137	1 × 10^8^	1 × 10^6^	113.500625
P12	ATLL	18.73	nd	1 × 10^5^	107.50451
P13	ATLL	nd	nd	1 × 10^4^	87.7595599
ND1	n/a	0	0	0	BG
ND2	n/a	0	0	0	BG

^*a*^ n/a represents healthy donor. nd represents not determined. BG represents background levels.

### Macaques experimentally infected with HTLV-1 or HTLV-2 have envelope-specific ADCC activity

**Fig 3:**
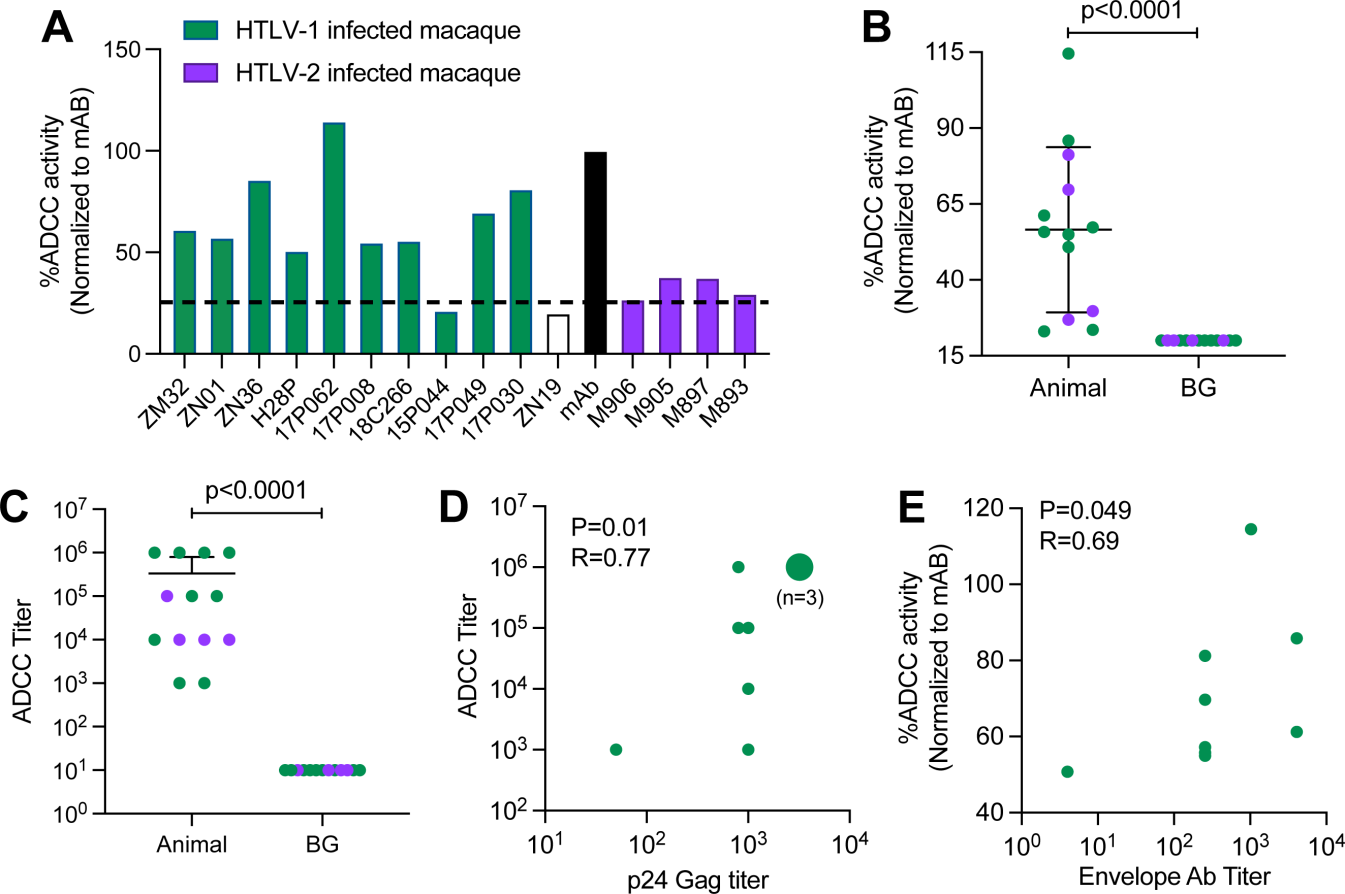
Envelope-specific ADCC activity in plasma from infected macaques. (**A**) ADCC killing activity from HTLV-1-infected macaques (green bars), HTLV-2-infected macaques (purple bars), or uninfected control (ZN19; white bar). The dotted line indicates the background level. (**B and C**) Comparison of (**B**) ADCC activity and (**C**) ADCC titer in HTLV-infected macaques from week 16 post-infection with background (BG). (**D**) Correlation between ADCC Ab titer and HTLV-1 p24 (Gag) Ab titer. (**E**) Correlation between ADCC activity and envelope Ab titer. Data shown in (**B and C**) were analyzed with two-tailed Mann-Whitney test. Horizontal and vertical bars denote mean and SD, respectively. Data shown in (**D and E**) were analyzed with two-tailed Pearson’s and two-tailed Spearman’s correlation test, respectively. Here, HTLV-1 and HTLV-2 samples are shown in green and brown, respectively.

Macaques are susceptible to HTLV-1A and HTLV-2 infection (33–35), and depletion of NK and CD8^+^ cells or monocytes, CD8^+^, and NK cells prior to experimental exposure to virus results in a robust infection and early seroconversion (36, 37). Thus, we tested plasma samples from experimentally infected macaques or uninfected controls to determine whether HTLV-positive macaques develop antibodies capable of inducing envelope-specific ADCC activity with our newly developed system (Table 2). Plasma samples came from previously published studies and were selected based on measurement of PVL and high HTLV antigen reactivity (33, 36, 37). In plasma samples from HTLV-1-infected macaques, we measured envelope-specific ADCC activity ranging from 21% to 114% when normalized with mAb (Fig. 3A; Table 2). Significant ADCC activity and antibody titer were observed compared to background levels (Fig. 3B and C). We excluded 15P044 from further analysis since the ADCC activity was below the background level. Interestingly, in HTLV-1-infected macaques, we further observed a significant positive correlation of ADCC titer with both HTLV-1 p24Gag (Fig. 3D) and envelope (Fig. 3E) antibody titers. Of note, we observed cross-reactive envelope-specific ADCC activity from plasma of HTLV-2-infected macaques (Fig. 3A; Table 2), with a range of ADCC antibody titers from 10^4^ to 10^5^, similar to what was measured in plasma from HTLV-1-infected macaques (Table 2). Since cross-reactivity has been observed between HTLV-1 and HTLV-2 envelope antibodies (38), this result was not unexpected. Due to the low numbers of animals, no correlation between ADCC activity, ADCC antibody titer, or proviral load was found for HTLV-2 infection.

**TABLE 2:** HTLV-infected macaques

Animal ID^*a*^	Treatment	Virus	Week post-infection	HTLV-1 p24Gag antibody titer	HTLV-1 gp46 envelope antibody titer	HTLV proviral DNA^*b*^	ADCC titer	Normalized killing
ZM32	M-T807R1	HTLV-1_WT_	18	3,200	4,096	<1	1 × 10^6^	61.24
ZN01	M-T807R1	HTLV-1_WT_	18	3,200	256	<1	1 × 10^6^	57.25
ZN36	M-T807R1	HTLV-1_WT_	18	800	4,096	<1	1 × 10^5^	85.86
H28P	IgG	HTLV-1_WT_	18	50	4	<1	1 × 10^3^	50.79
17P062	M-T807R1/Clodrosome	HTLV-1_WT_	16	1,000	1,024	38	1 × 10^3^	114.52
17P008	M-T807R1/Clodrosome	HTLV-1_WT_	16	1,000	256	6	1 × 10^4^	54.96
18C266	M-T807R1/Clodrosome	HTLV-1_WT_	16	1,000	256	5	1 × 10^5^	55.8
15P044	M-T807R1/Clodrosome	HTLV-1_WT_	16	800	64	9	1	21.33
17P049	M-T807R1/Clodrosome	HTLV-1_WT_	16	3,200	256	<1	1 × 10^6^	69.68
17P030	M-T807R1/Clodrosome	HTLV-1_WT_	16	800	256	58	1 × 10^6^	81.24
ZN19	M-T807R1	None	16	0	0	0	1	19.21
M906	Untreated	HTLV-2	12	n/a	n/a	18	1 × 10^4^	26.91
M905	Untreated	HTLV-2	12	n/a	n/a	54	1 × 10^4^	37.94
M897	Untreated	HTLV-2	12	n/a	n/a	12	1 × 10^5^	37.59
M893	Untreated	HTLV-2	12	n/a	n/a	22	1 × 10^4^	29.74

^*a*^ Plasma from animals listed here came from previously published studies [Moles et al. (37), Gutowska et al. (36), and Gordon et al. (33)].

^*b*^ Proviral load is given as copies per 1 million PBMCs.

### Changes in ADCC activity throughout the course of infection with different HTLV-1 infectious clones

**Fig 4:**
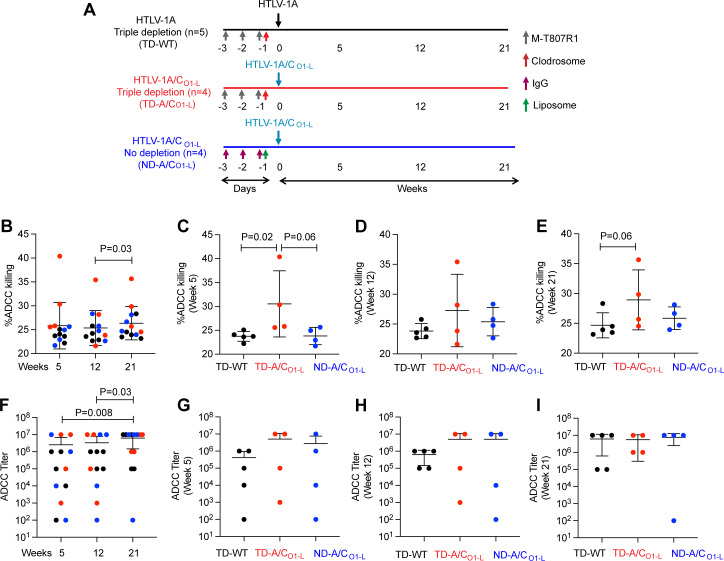
Evaluation of ADCC activity and titer in plasma from HTLV-1A versus HTLV-1A/C_O1-L_ infected macaques. (**A**) Schematic diagram of the study design for infection of macaques with HTLV-1A (black, *n* = 5) or HTLV-1A/C_O1-L_ (red and blue, *n* = 4 each). The HTLV-1A triple depletion group and HTLV-1A/C_O1-L_ triple depletion group received M-T807R1 antibodies (gray arrows) for three consecutive days before viral challenge and clodrosome 1 day (red arrow) before viral challenge. The HTLV1-A/C_O1-L_ no depletion group respectively received IgG (purple arrows) and Liposome (green arrow) at the same time point. The black or blue arrow indicates intravenous injection of HTLV-1A or HTLV1-A/C_O1-L_ virus-producing lethally irradiated cells, respectively. (**B**) Comparison of ADCC killing activity in all animals over the course of the study. (**C–E**) Comparison of ADCC killing activity among triple depleted HTLV-1A (TD-WT, black), triple depleted HTLV1-A/C_O1-L_ (TD- A/C_O1-L_, red), and non-depleted HTLV1-A/C_O1-L_ (ND- A/C_O1-L_, blue) animals at weeks (**C**) 5, (**D**) 12, and (**E**) 21 post-infection. (**F**) Comparison of ADCC titer in all animals over the course of the study. (**G–I**) Comparison of ADCC titer among triple depleted HTLV-1A (TD-WT, black), triple depleted HTLV1-A/C_O1-L_ (TD- A/C_O1-L_, red), and non-depleted HTLV1-A/C_O1-L_ (ND- A/C_O1-L_, blue) animals at weeks (**G**) 5, (**H**) 12, and (**I**) 21 post-infection. Data shown in (**B and F**) were analyzed with two-tailed Wilcoxon signed-rank test and (**C–E, G–I**) were analyzed with two-tailed Mann-Whitney test. Horizontal and vertical bars denote mean and SD, respectively.

HTLV-1 is genetically classified into seven subtypes, with subtype A (HTLV-1A) being the most globally widespread and subtype C (HTLV-1C) being predominant in Australia and Oceania (39). The highest nucleotide divergence between HTLV-1C and HTLV-1A is primarily in the 3′-end of the virus encoding several regulatory genes (40, 41). We and others have previously shown that the 3′ regulatory genes play an important role in viral persistence and transmission, reviewed in references 42–46. Next, we infected rhesus macaques with HTLV-1A or a chimeric HTLV-1A/C virus encoding the HTLV-1A envelope gene, constructed by inserting the HTLV-1C 3′-end into the HTLV-1A backbone, to explore differences in viral infectivity, cytokine responses, viral pathogenesis, changes in cellular populations, and inflammation status between the two HTLV-1 viruses (Sarkis, unpublished data) (Fig. 4A; Fig. S1C). Here, we used our new assay to investigate ADCC activity and antibody titer in the above longitudinal study to determine whether ADCC responses differed between the infected macaque groups. ADCC activity and titer were measured using plasma samples from weeks 5, 12, and 21 post-infection (Fig. 4B through I) in three groups of animals: (1) CD8^+^, NK cell, and monocyte triple depleted (TD) animals infected with HTLV-1A wild type (WT) virus (TD-WT, shown in black, *n* = 5); (2) CD8^+^, NK cell, and monocyte triple depleted animals infected with chimeric HTLV-1A/C virus (TD-AC_O1-L_, shown in red, *n* = 4); and (3) non-depleted (ND) animals infected with chimeric HTLV-1A/C virus (ND-AC_O1-L_, shown in blue, *n* = 4). We observed that, by week 5 post-infection, the depleted cell populations returned to baseline or above, independent of the virus subtype (Sarkis, unpublished). Here, we detected a significant increase in the percent of ADCC killing and ADCC antibody titers from week 12 to week 21 in plasma from all infected animals (*P* = 0.03; Fig. 4B and F). To investigate if there was a difference between treatment or virus, we next evaluated ADCC killing activity for each group individually at week 5 (Fig. 4C), week 12 (Fig. 4D), and week 21 (Fig. 4E). Trends for a higher percent of ADCC killing were observed in the plasma of triple depleted animals infected with HTLV-1A/C (TD-A/C_O1-L_, red) compared to HTLV-1A (TD-WT, black) at week 5 (*P* = 0.02) and week 21 (*P* = 0.02 and *P* = 0.06, respectively; Fig. 4C and E). At week 12, the ADCC activity was comparable for both viruses (Fig. 4D). We also measured an increase in ADCC antibody titer over time in all infected animals independent of treatment or virus (Fig. 4G through I). These findings are consistent with a persistent ongoing infection. Together, these results demonstrate that this cell line-based assay system can be used to quantify HTLV envelope-specific ADCC activity in the macaque model.

### Correlation of HTLV-1 infection induced immune responses to ADCC responses

**Fig 5:**
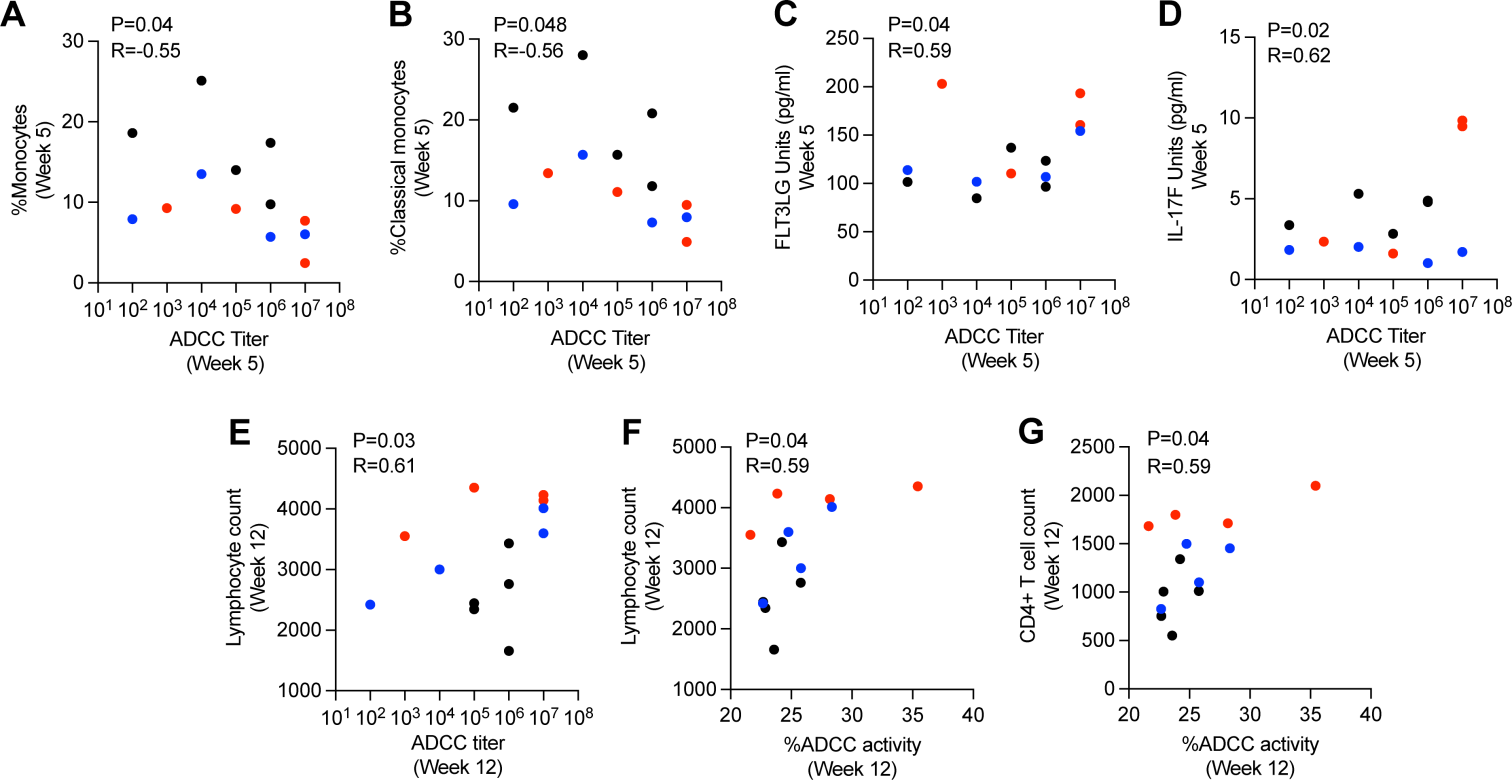
Correlation of ADCC activity at week 5 and week 12 post-infection with immune responses. (**A and B**) Negative correlations between ADCC titer and the frequency of (**A**) monocytes or (**B**) classical monocytes. (**C and D**) Positive correlations between ADCC titer and (**C**) FLT3LG or (**D**) IL17F cytokine plasma levels. (**E and F**) Correlation of total lymphocyte count with ADCC titer and ADCC killing activity, respectively. (**G**) Trend of positive correlation between ADCC killing activity (%) and total CD4^+^ cell count. Here, TD-WT (black), TD-ACO1-L (red), or ND-ACO1-L (blue). Data shown in (**A–E**) were analyzed with two-tailed Pearson’s correlation test and (**F and G**) were analyzed with two-tailed Spearman’s correlation test.

To investigate the interplay of ADCC activity with other immune responses, we performed a correlation analysis between ADCC and cell populations (Table S1) or cytokines (Table S2) from a longitudinal HTLV-1 study of experimentally infected macaques. At week 5 post-infection, monocyte frequencies (Fig. 5A) and classical monocyte frequency (Fig. 5B) negatively correlated with ADCC titer, respectively. This negative correlation is consistent with a report that monocytes inhibit NK cell-mediated ADCC (47) and that CD16 is important for monocytes to exert ADCC (48). Of the 40 cytokines analyzed by proximity extension assay (Olink) proteomic technology, IL-17F and FLT3LG showed a positive correlation to ADCC titer (Fig. 5C and D), suggesting that these cytokines may help NK cells and subsequently promote ADCC. It is known that FLT3LG supports the development of NK and B cells and dendritic cells (49), and IL-17F is produced by NK and other innate cells (50).

We further investigated the correlation between ADCC activity and different immune parameters at week 12 post-infection. ADCC titer and killing activity positively correlated with lymphocyte count (Fig. 5E and F). There was a positive correlation of ADCC killing to CD4^+^ T count (Fig. 5G). At week 21 post-infection, no correlations of ADCC titer or killing activity with other immune parameters were found (data not shown). Together, these results demonstrate that ADCC activity is influenced by immune responses generated by HTLV infection over time and manipulation of these non-neutralizing antibodies might modulate the host immune response to the virus. However, clearly more studies with a larger number of animals are needed to determine what parameters are important for influencing ADCC activity and its importance in viral HTLV clearance and the interplay between cytokine levels, disease progression, and envelope-specific ADCC. Importantly, this new assay system will allow this line of studies to be performed.

## DISCUSSION

A better understanding of host control of viral persistence is necessary to develop effective vaccines and therapeutics for pathogens that cause chronic infection and impair host immunity. The development of HTLV-associated diseases is greatly influenced by the immune response to the virus (51–53). While the majority of HTLV-1-infected individuals remain asymptomatic, about 10% will develop high morbidity and HTLV-1-associated disease. Almost all cases of adult T-cell leukemia and infective dermatitis can be attributed to virus acquisition through mother-to-child transmission (MTCT), as well as approximately 30% of HAM/TSP cases (54–56). Development of HAM/TSP is strongly associated with sexual transmission and blood transfusion (54). However, the contribution of host immune responses to the differences in disease development is poorly understood.

ADCC plays a major role in controlling infection, particularly for viruses that spread via cell-to-cell transmission and proliferation of infected cells. High ADCC-inducing antibodies have been detected in sera from HTLV-infected individuals as shown here and in other studies (21, 22). The HTLV-1 gp46 envelope glycoprotein has been identified as the primary Ab target (20, 24). Further, a few reports suggesting that reduced ADCC activity does not correlate with viral DNA level but correlates with disease progression have been published (14, 20, 57) and that anti-gp46 specific neutralizing and ADCC-inducing antibodies protected against HTLV-1 infection in a rat MTCT model and hu-PBL-NOG mouse model (58, 59).

Studies testing ADCC-inducing HTLV-1 antibodies have relied on HTLV-1 immortalized or transformed cells as targets. There are several limitations to this approach. The HTLV-1 immortalized or transformed cells are not resistant to direct NK cell lysis, and there is thus background killing independent of ADCC activity. This level varies among effector cells, and it is therefore difficult to develop a high-throughput system as several effector-to-target ratios must be assessed before ADCC-induced killing can be measured above background (60). In addition, these target cells produce infectious virus that could alter cell signaling and/or function *in vitro* (61). Indeed, it has been shown that infected cells induce spontaneous proliferation of CD8^+^ and NK cells when PBMCs from infected individuals are cultured *ex vivo* (62–64). Finally, it has been shown that viral proteins including p8 can be transferred to uninfected cells through cellular conduits (65) and quantitative analysis indicates that, within 5 minutes of co-culture, 5% of recipient cells contain p8 (66).

**Fig 6:**
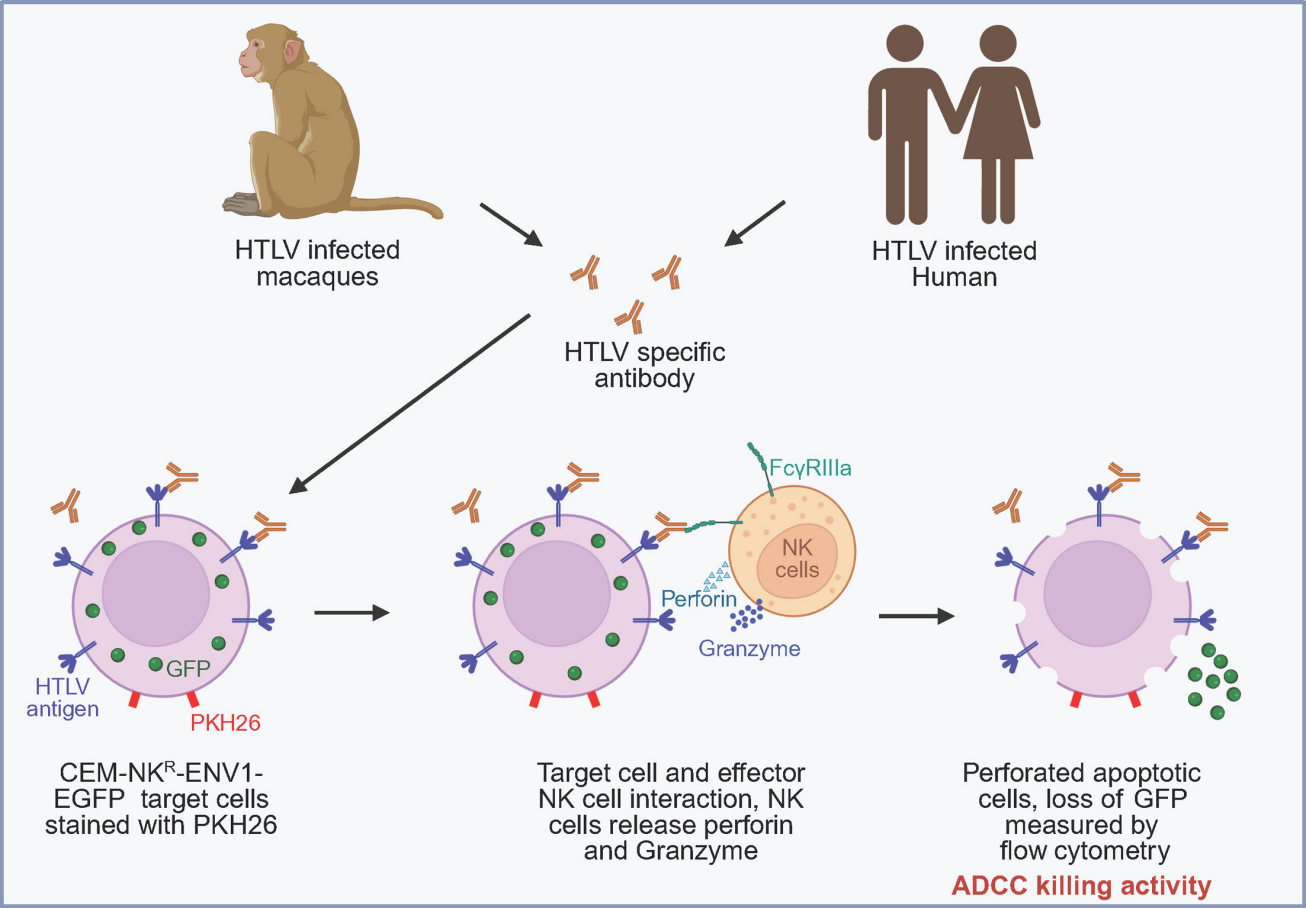
HTLV-specific antibody-dependent cellular cytotoxicity. Plasma from HTLV-infected macaques and human patients binds to HTLV antigen on the surface of CEM-NK^R^-ENV1-EGFP target cells, which constitutively express GFP and plasma membrane labeled with PKH26. NK cells interact with the antibody-bound, HTLV-infected CEM-NK^R^-ENV1-EGFP target cells through the FcγRIIIa receptor. This interaction triggers the release of perforin and granzyme from NK cells, forming pores in the target cells and leading to GFP release. The loss of GFP in PKH26-positive cells represents the fraction of cells killed through an antibody-dependent mechanism. This figure was created by F. Bhuyan with BioRender.com.

Here, we developed a sensitive high-throughput assay to specifically measure HTLV envelope antigen-specific ADCC (Fig. 6). The advantage of our system is that we incorporate NK-resistant cells expressing only the HTLV-1 envelope protein, with HTLV-1 and HTLV-2 cross-reactive epitopes. Using this assay, we quantified envelope-specific antibody-killing activity and ADCC titers in the plasma of HTLV-1-infected humans and experimentally infected macaques. In macaques, ADCC titer correlated with p24Gag antibody titer (Fig. 3D) and ADCC activity correlated with envelope antibody titer (Fig. 3E). Next, we used the assay to measure ADCC activity and antibody titers in our longitudinal HTLV-infected macaque model (Fig. 4; Table 2). Interestingly, we found that ADCC killing activity and antibody titers increased over time, suggesting that ADCC develops during persistent infection. In depleted animals, infection with HTLV-1A/C trended to a higher level of ADCC killing compared to HTLV-1A-infected macaques or to replete animals infected with HTLV-1A/C (Fig. 4C) despite both viruses encoding the HTLV-1A envelope (Fig. S1C). This is consistent with HTLV-1A/C infection inducing a higher frequency of pro-inflammatory myeloid cells and neutrophils compared to HTLV-1A in depleted animals (Sarkis, unpublished). Of note, we recently demonstrated that the depletion of monocytes, NK cells, and CD8^+^ cells prior to infection with HTLV-1 allowed a robust infection of all exposed animals (36). While correlations between ADCC killing activity and antibody titers were seen for cell populations and specific cytokines at individual time points in the study, no correlation with p24Gag antibody titers or proviral load was observed in these samples. These results suggest that although triple depletion results in a more robust infection, it may alter the antibody response to the virus. More studies are needed to investigate the interplay between cell populations in the generation of the quality and magnitude of the antibody response to HTLV.

One limitation of the study is the low frequency of HTLV-1 antigen expression in the CEM-NK^R^-ENV1a-EGFP cell line. We observed that, while the CEM-NK^R^-ENV1a-EGFP cell line elicited specific killing responses, the CEM-NK^R^-ENV1b-EGFP cell line did not show any specific killing, suggesting that not all transfections will result in generating a target cell line suitable for this assay. Although efforts were made to enhance the frequency of antigen expression in these cells, they were unsuccessful, even after FACS sorting. In this study, we observed that the low-frequency HTLV-1 env-expressing cells can generate specific ADCC responses. Further techniques such as serial dilution might help to generate a clonal cell line and might give more robust ADCC responses.

Thus, our newly developed ADCC assay fills gaps in the field, providing a high-throughput system to accurately measure HTLV envelope-specific ADCC for use in clinical, therapeutic, and vaccine studies for various model systems.

## MATERIALS AND METHODS

### Plasmids

The HTLV-1A envelope cDNA was synthesized and cloned into the pUC-Zeo vector (Azenta Life Sciences, Burlington, MA). An HA-tag was inserted at the 3′-end of the envelope sequence for western blot detection. The KpnI site in the C-terminal coding region of the envelope was destroyed without changing the amino acid sequence using QuikChange Lightning (Agilent, Santa Clara, CA) as described by the manufacturer. The KpnI-XbaI fragment (1,517 base pairs) was then placed in the lentiviral vector, pLVX-IRES-Puro (Takara Bio, Inc., Madison, WI).

### Cell lines and establishment of stable CEM-NK^R^-ENV1-EGFP cells

HTLV-1 transformed cell lines MT2 and C91PL and the B cell lines 729.6 and 729-D26 (HTLV-1A) (35) were grown in RPMI supplemented with 10% heat inactivated fetal bovine serum and 100 U/mL penicillin/streptomycin (R10 medium). To assess HTLV-1A envelope expression, we transiently transfected 293FT cells using LipoD293 (SignaGen Laboratories, Frederick, MD) with the envelope lentiviral expression construct following the manufacturer’s instructions. Once proper expression was determined by western blot analyses using anti-HA (Cell Signaling Technology, Inc., Danvers, MA) and anti-HTLV-1 gp-46 antibodies (Creative Biolabs, Shirley, NY), lentiviral stocks were produced using Lenti-X Packaging Single Shots and the pLVX-Env1 vector was transfected into Lenti-X 293T cell line as described by the manufacturer’s instructions (Takara Bio, Inc., Madison, WI).

CEM-NK^R^-EGFP cells were transduced with lentiviral particles, as described previously (67). Twenty-four hours after transduction, stable cell lines were produced by culturing cells in DMEM supplemented with 5% fetal bovine serum and 0.1 µg/mL puromycin at a density of 1 cell/100 µL in 96-well plates.

### HTLV-1 proviral load

HTLV-1 proviral load (PVL) was measured by real-time PCR using 5 ng/µL genomic DNA and TaqMan Universal PCR Master Mix (Applied Biosystems, Foster City, CA) with the Rotor-Gene Q system (Qiagen, Germantown, MD). Standard curves were generated by amplification of a RNase P gene fragment from HTLV-1 negative genomic DNA isolated from PBMCs with the use of Taqman RNase P Detection Reagents VIC (Applied Biosystems, Foster City, CA) and a fragment of the HTLV-1 pX region from the HTLV-1 wild-type molecular clone plasmid (35). The primer and probe sequences for the pX gene were as follows: 5′-CGGATACCCAGTCTACGTGTT-3′, 5′-CAGTAGGGCGTGACGATGTA-3′, and 3′-FAM/CTGTGTACAAGGCGACTGGTGCC-3′ (68). The reaction conditions were one cycle of 2 minutes at 50°C, followed by one cycle of 10 minutes at 95°C, 40 cycles of 15 seconds at 95°C, then 60 seconds at 60°C. HTLV-1 provirus DNA levels (PVL) were calculated by the following formula: (copies of HTLV-1 (pX)/(copies of RNase P/2) = copies/cell).

### Effector cells and plasma samples

Human peripheral blood mononuclear cells (PBMCs) were used as effector cells. PBMCs were separated from blood of healthy human donors by density gradient centrifugation using Ficoll Plaque (GE Healthcare, Silver Spring, MD). All Indian rhesus macaque (*Macaca mulatta*) samples stored at −80°C were obtained from previously published studies (33, 36, 37). HTLV-1 uninfected healthy volunteers screened at the NIH Clinical Center (Bethesda, MD, USA) were evaluated as normal donors (ND). Sera from HTLV-1-infected individuals were prepared from blood samples and stored in a −80°C freezer until use.

### ADCC flow cytometric assay

ADCC activity was assessed using CEM-NK^R^-ENV1-EGFP cells or CEM-NK^R^-EGFP cells (NIH HIV Reagent Program, Bethesda, MD) that constitutively express GFP as targets. Target cell membranes were labeled with PKH26 following the manufacturer’s recommendation (Sigma-Aldrich, St. Louis, MO). Plasma samples, heat inactivated at 56°C for 30 minutes, were serially diluted (seven 10-fold dilutions starting at 1:10) and 100 µL was added to wells of a 96-well V-bottom plate (Millipore Sigma, St. Louis, MO). Anti-HTLV-1-gp46 monoclonal antibody PRH7a was used as a positive control and anti-HIV gp120 antibody NCI09 was used as a negative control (18). Briefly, one million target cells were incubated with plasma or antibodies for 1 hour at 37°C. A total of 5,000 target cells (50 µL) and 250,000 human PBMCs (50 µL) were added as effectors to each well to give an effector/target (E/T) ratio of 50:1. The plate was incubated at 37°C for 2 hours followed by two phosphate-buffered saline (PBS) washes. The cells were resuspended in 200 µL of a 2% PBS–paraformaldehyde solution and acquired on a BD FACSymphony equipped with a high-throughput system (BD Biosciences, Franklin Lakes, NJ). Specific ADCC activity was measured by loss of GFP from the target cells. Target and effector cells cultured in the presence of R10 medium were used as background. Normalized ADCC activity was calculated as: (ADCC activity in the presence of plasma − background)/(ADCC activity in the presence of PRH7a − background) × 100. The normalization was done to minimize plate-to-plate and experiment-to-experiment variation of the assay. The ADCC endpoint titer is defined as the reciprocal dilution at which the percent ADCC activity was greater than the mean percent ADCC activity of the background wells containing medium only with target and effector cells, plus three standard deviations. A similar method was used to measure ADCC activity in MT2 and C91PL cells with the following modifications. MT2 and C91PL cells were labeled with PKH26 and carboxyfluorescein succinimidyl ester (CFSE) (Thermo Fisher Scientific, Waltham, MA). Target cells were cultured with PRH7a or IgG control.

### Plasma HTLV-1 envelope titer

HTLV-1A envelope antibodies were measured by ELISA. ELISA 96-well plates (Thermo Fisher Scientific, Waltham, MA) were coated with 50 ng/well of HTLV-1 gp46 protein in 50 mM sodium bicarbonate buffer (pH 9.6) and incubated overnight at 4°C. Plates were washed twice with PBS blocked with 5% nonfat dry milk (Biorad, Hercules, CA) for 2 hours at room temperature. Wash wells with PBS once before adding 50 µL of plasma samples (serially diluted) to the wells. Plates were covered and incubated overnight at 4°C, washed four times with PBS-Tween 20 (0.05%; PBST), and incubated with 50 µL/well anti-human HRP (1:10,000 in PBST) or anti-Non-Human Primate HRP (1:10,000 in PBST) for 1 hour covered at room temperature. The plates were washed four times with PBST. Plates were developed using TMB (3,3′,5,5′ tetramethylbenzidine) ultra substrate (Thermo Fisher Scientific, Waltham, MA) to all wells. The reaction was stopped with TMB Stop Solution (SeraCare, Gaithersburg, MD) and the plate was read at 450 nm on a Molecular Devices E-max plate reader.

### Virus inoculation and treatments in the macaque model

Rhesus macaques uninfected with SIV/SHIV as demonstrated by several consecutive negative PCR and seronegative for simian T-cell lymphotropic virus 1 at the initiation of the study were randomized into groups based on their sex, age, weight, and their prior enrollment in other studies. Animals in groups designated as triple depletion (TD) were treated for three consecutive days (days −3, −2, and −1) with M-T807R1, an anti-CD8 monoclonal antibody targeting CD8^+^ lymphocytes, and NK cells. In addition, on day −1, a single dose of Clodrosome to deplete monocytes/macrophages was administered. Animals used as controls and belonging to the group designated as non-depleted (ND) were treated for three consecutive days (days −3, −2, and −1) with the isotype control antibody, along with a single dose of empty Liposome (Encapsome). Both isotype control and M-T807R1 antibodies were purchased from the NHP Reagent Resource Program (University of Massachusetts Medical School, Worcester, MA). Clodrosome and Encapsome were purchased from Encapsula NanoSciences LLC, Brentwood, Tennessee. All treatments were administered intravenously at 5 mg/kg/dose/day prior to the intravenous inoculation of 1.5 × 10^8^ lethally γ-irradiated 729.6 B lymphoblastoid HTLV-1A and HTLV-1A/C_O1-L_ producer cell lines (36) (Sarkis, unpublished). Blood samples were collected before the start of the study (baseline), day 0, week 5, week 12, and week 21.

### Cell populations and cytokine profiles

For whole blood phenotyping, fresh EDTA whole blood (100 µL) was stained and analyzed as previously described (36). Fluochrome-conjugated antibodies include: FITC anti-CD8 (cloneDK25; EMB Millipore Corp.), BB700 anti-CD14 (clone MSE2; BD Biosciences), PE-Cy5 anti-CD95 (clone DX2; BioLegend), PE-Cy7 anti-CD159 (NKG2a) (clone Z199; Beckman Coulter), APC anti-CD66abce (clone TET2; Miltenyi Biotec), Alexa 700 anti-CD3 (clone SP34-2; BD Biosciences), APC-Cy7 anti-CD11b (clone D12; BD Biosciences), BV421 anti-CD16 (clone 3G8; BD Biosciences), BV570 anti-CD20 (clone 2H7; BioLegend), BV605 anti-CD194 (CCR4) (clone 1G1; BD Biosciences), BV750 anti-CD4 (clone L200; BD Biosciences), BV786 anti-CD45 (clone D058-1283; BD Biosciences), BUV496 anti-CD28 (clone CD28.2; BD Biosciences), BUV563 anti-CD49d (clone 9F10; BD Biosciences), BUV661 anti-HLA-DR (clone G-46–6; BD Biosciences), BV711 anti-CD11c (clone B-ly6; BD Biosciences), BV650 anti-CD123 (clone 7G3; BD Biosciences), and BUV805 anti-CD8 (clone SK1; BD Biosciences). Blue LIVE/DEAD viability dye (Thermo Fisher Scientific, Waltham, MA) was used to exclude dead cells. Flow cytometry acquisitions were performed on a FACSymphony A5 and examined using FACSDiva software (BD Biosciences) by acquiring all stained cells. Data were further analyzed using FlowJo v10.1 (TreeStar, Inc., Ashland, OR).

### Proximity extension assay (PEA) on plasma samples

The Olink Target 48 Cytokine panel* (Olink Proteomics AB, Uppsala, Sweden) was used in accordance with the manufacturer’s protocols for protein quantification. This specific PEA methodology enables the concurrent assessment of 45 distinct analytes. Biomark HD system by Fluidigm (Olink Signature Q100 instrument) was used for detection and quantification (69). Data validation was conducted with the Olink NPX Signature software specifically designed for the Olink analysis: the application was used to import data from the Olink Signature Q100 instrument and process the data. Data normalization procedures were executed employing internal extension control and calibrators, thereby effectively mitigating any inherent intra-run variability. The cytokines TSLP, IFNγ, CCL7, IL1β, IL2, IL4, IL10, and IL27 were below the level of detection and not used in the analysis. The assay output is reported in picograms per milliliter (pg/mL), established upon a robust 4-parameter logistic (4-Pl) fit model, thereby ensuring precise absolute quantification. Comprehensive insights into the assay’s validation parameters, encompassing limits of detection, intra- and inter-assay precision data, and related metrics are available at www.olink.com.

### Statistical analysis

The two-tailed Wilcoxon signed-rank test or two-tailed Mann-Whitney test was used to compare continuous factors between two paired or unpaired groups, respectively. Correlation analyses were performed using the non-parametric two-tailed Spearman-rank correlation test. The normal distribution of the data was analyzed with the D'Agostino & Pearson test and, in the case of normal distribution, the two-tailed Pearson’s correlation method. Since this research was conducted as exploratory, all *P* values are reported as nominal values without adjusting for multiple comparisons. Statistical analysis was performed using GraphPad Prism version 10.3 (GraphPad Software, Inc., La Jolla, CA). As indicated, data are expressed as mean with SD. *P* values of less than 0.05 were considered significant. *P* values are indicated on graphs. The statistical test used is indicated in the figure legend.

## Data Availability

Raw data have been provided in supplemental figures and tables.

## SUPPLEMENTAL MATERIAL

Figure S1 jvi.02268-24-s0001.tiff Western blot analysis and schematic.

Supplemental legends jvi.02268-24-s0002.pdf Legends for supplemental tables and figure.

Table S1 jvi.02268-24-s0003.xlsx ADCC activity and cell populations from a longitudinal study of HTLV-1A or HTLV-1A/C infected rhesus macaques.

Table S2 jvi.02268-24-s0004.xlsx Cytokine profile from longitudinal study of HTLV-1A or HTLV-1A/C infected rhesus macaques.

Tables S3 to S7 jvi.02268-24-s0005.xlsx ADCC activity, antibody titers, and cell populations for Fig. 1 to 5.

Table S8 jvi.02268-24-s0006.xlsx HTLV-1 proviral load.

ASM does not own the copyrights to Supplemental Material that may be linked to, or accessed through, an article. The authors have granted ASM a non-exclusive, world-wide license to publish the Supplemental Material files. Please contact the corresponding author directly for reuse.
