# Supplementary figures and images for: Development and optimization of human T-cell leukemia virus-specific antibody-dependent cell-mediated cytotoxicity (ADCC) assay directed to the envelope protein
Source: J Virol. 2025 Mar 28;99(5):e02268-24. doi: 10.1128/jvi.02268-24 (PMC12090781; doi:10.1128/jvi.02268-24)

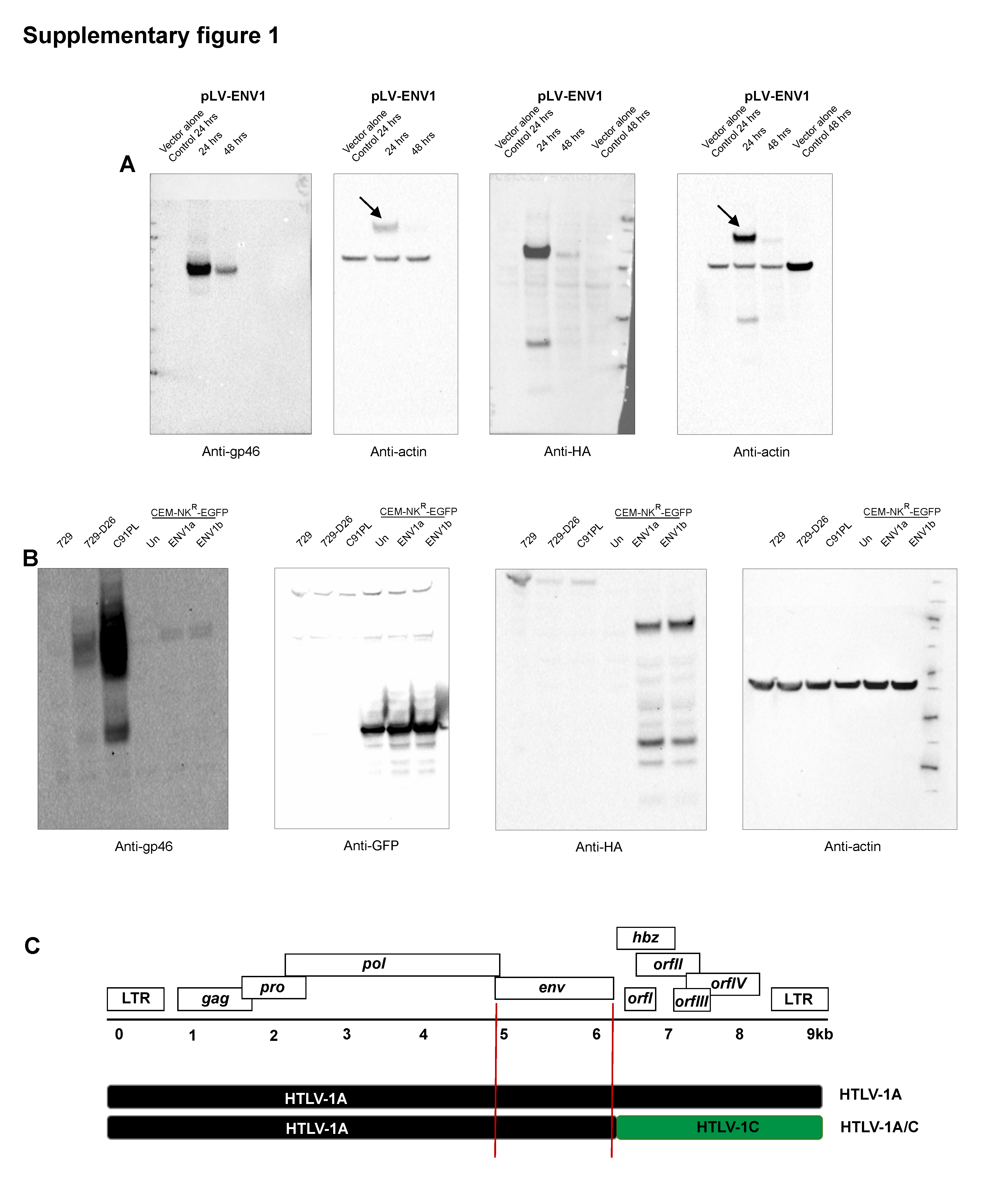

Supplement: Figure S1 — Western blot analysis and schematic. [file jvi.02268-24-s0001.tiff]
