# Supplementary material for: Development and optimization of human T-cell leukemia virus-specific antibody-dependent cell-mediated cytotoxicity (ADCC) assay directed to the envelope protein
Source: J Virol. 2025 Mar 28;99(5):e02268-24. doi: 10.1128/jvi.02268-24 (PMC12090781; doi:10.1128/jvi.02268-24)
Supplement: Supplemental legends — Legends for supplemental tables and figure. [file jvi.02268-24-s0002.pdf]

SUPPLEMENTAL INFORMATION

FIG S1. **(A)** Western blot analysis of whole cell lysates from 293T cells transient transfected with pLV-ENV1 expression construct or vector control for time indicated. After staining with antibody to either Anti-pg-46 (first panel) or Anti-HA (third panel) antibodies, membranes were stripped and re-probed with Anti-actin for loading control. Arrows indicate bands not completely stripped from first probing. **(B)** Western blot analysis of cell lysates from control HTLV-1 negative cells 729 or HTLV-1^{+} cells 729-D26 and C91PL or the CEM-NKR-EGFP parental (Un) or envelope expressing ENV1a and ENV1b. Membranes from Fig 1C were stripped and re-probed with Anti-GFP or Anti-actin antibodies for loading controls. **(D)** A schematic diagram of HTLV-1 provirus for the HTLV-1A and chimeric HTLV-1A/C constructs. The viral gene structure is displayed above the kilobase marker. Nucleotide sequences corresponding to HTLV-1A are in solid gray and nucleotide sequences from HTLV-1C in stippled green.

TABLE S1. ADCC activity and cell populations from a longitudinal study of HTLV-1A or HTLV-1A/C infected rhesus macaques.

TABLE S2. Cytokine profile from longitudinal study of HTLV-1A or HTLV-1A/C infected rhesus macaques.

TABLE S3. ADCC in HTLV-1 Transformed cells (Figure 1).

TABLE S4. ADCC Activity and Antibody Titers for Figure 2.

TABLE S5. ADCC Activity and Antibody Titers for Figure 3.

TABLE S6. ADCC Activity and Titer for Figure 4.

TABLE S7. ADCC Titer and cell populations for Figure 5.

TABLE S8. HTLV-1 Proviral Load.
